# Interleukin-6 and C-reactive protein as prognostic biomarkers in metastatic colorectal cancer

**DOI:** 10.18632/oncotarget.12601

**Published:** 2016-10-12

**Authors:** Maria Thomsen, Christian Kersten, Halfdan Sorbye, Eva Skovlund, Bengt Glimelius, Per Pfeiffer, Julia S. Johansen, Elin H. Kure, Tone Ikdahl, Kjell Magne Tveit, Thoralf Christoffersen, Tormod Kyrre Guren

**Affiliations:** ^1^ Department of Oncology, Oslo University Hospital, Oslo, Norway; ^2^ Institute of Clinical Medicine, University of Oslo, Oslo, Norway; ^3^ Department of Oncology, Southern Hospital Trust, Kristiansand, Norway; ^4^ Department of Oncology, Haukeland University Hospital, University of Bergen, Bergen, Norway; ^5^ Department of Public Health and General Practice, Norwegian University of Science and Technology, Trondheim, Norway; ^6^ Department of Immunology, Genetics and Pathology, Uppsala University, Uppsala, Sweden; ^7^ Department of Oncology, Odense University Hospital, Institute of Clinical Research, University of Southern Denmark, Odense, Denmark; ^8^ Department of Oncology, Herlev and Gentofte Hospital, Copenhagen University Hospital, Herlev, Denmark; ^9^ Department of Cancer Genetics, Institute for Cancer Research, Oslo University Hospital, Oslo, Norway; ^10^ Akershus University Hospital, Nordbyhagen, Norway; ^11^ K.G. Jebsen Colorectal Cancer Research Centre, Oslo University Hospital, Oslo, Norway; ^12^ Department of Pharmacology, Institute of Clinical Medicine, Faculty of Medicine, University of Oslo, Oslo, Norway

**Keywords:** mCRC, IL-6, CRP, prognostic biomarker, survival

## Abstract

**Objectives:**

The aim was to explore the prognostic significance of IL-6 and markers of systemic inflammatory response (SIR), in particular C-reactive protein (CRP), in metastatic colorectal cancer (mCRC) patients, in the total study population and according to *RAS* and *BRAF* mutation status.

**Results:**

High levels of pretreatment serum IL-6 or CRP were associated with impaired outcome, in terms of reduced PFS and OS. Patients with low versus high serum IL-6 levels had median OS of 26.0 versus 16.6 months, respectively (*P* < 0.001). Stratified according to increasing CRP levels, median OS varied from 24.3 months to 12.3 months, (*P* < 0.001). IL-6 and CRP levels affected overall prognosis also in adjusted analyses. The effect of IL-6 was particularly pronounced in patients with *BRAF* mutation (interaction *P* = 0.004).

**Materials and Methods:**

IL-6 and CRP were determined in pre-treatment serum samples from 393 patients included in the NORDIC-VII trial, in which patients with mCRC received first line treatment. The effect of serum IL-6 and CRP on progression-free survival (PFS) and overall survival (OS) was estimated.

**Conclusions:**

High baseline serum consentrations of IL-6 or CRP were associated with impaired prognosis in mCRC. IL-6 and CRP give independent prognostic information in addition to *RAS* and *BRAF* mutation status.

## INTRODUCTION

Colorectal cancer (CRC) is worldwide the second most commonly diagnosed cancer in females and the third in males. Almost half of the patients diagnosed with CRC have or will develop distant metastases [1]. Metastatic CRC (mCRC) follows widely differing clinical courses, depending on genetic and non-genetic factors. A large body of evidence indicates that an inflammatory microenvironment is of decisive importance for the progression of tumors and the clinical outcome of cancers [2–6], including CRC [7]. It would be valuable to have easily accessible biomarkers for monitoring of the inflammatory status of the cancer, and several inflammation-based factors have been evaluated for their usefulness as prognostic biomarkers [6, 8, 9].

While critical mutations are the underlying drivers in oncogenesis [10], interaction between the transformed cells and the microenvironment is necessary for a cancer to evolve, progress, and metastasize [2, 11, 12]. Malignant cells may evade the many innate and adaptive defense mechanisms and eventually give rise to neoplasms, due to their ability to induce immune suppression and to control and redirect the functions of several of the stromal cells, shaping a symbiotic local environment [11] which basically takes the character of an inflammatory reaction, a recognized hallmark of cancer [3, 13]. Although the stroma is the arena for numerous opposing stimulatory and inhibitory mechanisms [14–17], the very existence of the tumor reveals its escape from the host defense, and many lines of evidence strongly suggest that the microenvironment is largely tumor-promoting at all stages of cancer [2, 11, 18].

A tumor-promoting inflammatory microenvironment is driven by interactions between the malignant cells, immune cells, and other stromal cells, which communicate through numerous growth factors, cytokines, and other locally active agents [18, 19]. These factors are essential for tumor growth, progression, and metastasis [11, 20]. In addition, some of the pro-inflammatory factors may exert effects that go beyond the local tumor, causing a state of systemic inflammation [11, 21], which is strongly implicated as an important cause of cancer-associated morbidity, in terms of pain, fatigue, functional disability, anorexia, and cachexia, as well as reduced treatment response and poor survival [5, 6, 22, 23].

Interleukin-6 (IL-6) is an important pro-inflammatory cytokine produced particularly by macrophages but also by other stromal cells such as neutrophils, cancer-associated fibroblasts and endothelial cells, as well as some cancer cells [24]. IL-6 exerts its effects on many cells [25, 26] through its receptor IL-6R with the associated gp130 and downstream mechanisms where JAK-STAT3 is a major pathway [19, 27]. IL-6 has a main role in sustaining chronic inflammation [28], and it is a potent inducer of hepatic synthesis of acute phase proteins, including C-reactive protein (CRP) [29]. Furthermore, several lines of evidence implicate IL-6 as a promoting factor in cancer [30–33] and suggest that IL-6 contributes to a chronic inflammatory and tumorigenic microenvironment in CRC [19, 34]. There are also reports showing that high IL-6 is associated with poor prognosis in CRC [35].

Measurable systemic effects of inflammation have been termed systemic inflammatory response (SIR). Different blood measures for SIR have been proposed [8, 9, 36, 37]. The relationship between these measures and tumorigenic inflammatory processes in CRC is not fully clarified. There is a need of further studies of the prognostic significance of different markers of SIR. The levels of SIR markers, such as the modified Glasgow Prognostic Score (mGPS), the derived neutrophil to lymphocyte ratio (dNLR), platelet levels, CRP levels, and high-sensitive CRP (hs-CRP) have all been found to predict cancer-specific survival [8, 37, 38]. Most of these studies come from cohorts of patients treated with curative surgery, and only a few reports focus on patients with metastatic cancer. It was recently reported that high hs-CRP levels predict poor survival in mCRC [38]. Little is known about the impact of inflammation in mutation subgroups in this cancer. The present study was based on a cohort from the NORDIC-VII study with patients receiving first-line treatment for mCRC [39]. The aim was to explore the prognostic role of serum IL-6 and CRP levels, in the total study population as well as in subgroups according to *RAS* and *BRAF* mutation status, and to study the value of prognostic inflammatory markers for use in daily clinical practice.

## RESULTS

### Baseline characteristics

Between May 2005 and October 2007, patients with untreated mCRC were enrolled in the NORDIC-VII study, randomized to first line treatment with standard Nordic FLOX, cetuximab and FLOX, or cetuximab combined with intermittent FLOX [39]. In the present study, the data were analyzed across the different treatment arms. Baseline demographics and clinical characteristics of the 393 patients included in this study are shown in Table 1.

**Table 1:** Baseline patient demographics and clinical characteristic, *n* = 393

Characteristics	Median (range)
**Age,** years	62.1 (24.1–74.9)
	**Number (%)**
**Sex**	
male	237 (60.3)
female	156 (39.7)
**WHO performance status**	
0	255 (64.9)
1	114 (29.0)
2	24 (6.1)
**Location**	
colon	233 (59.3)
rectum	160 (40.7)
**No.of metastatic site**	
1	123 (31.3)
> 1	270 (68.7)
**Primary tumor resected**	
no	95 (24.2)
yes	298 (75.8)
**Liver only**	
yes	82 (20.9)
no	311 (79.1)
**Alkaline phosphatase**	
normal	212 (53.9)
abnormal	181 (46.1)
**Prior adjuvant chemotherapy**	42 (10.7)

Analysis regarding tumor burden and site of metastases in relation to IL-6 and CRP show that the distribution of inflammatory markers was relatively similar in the different groups, see Supplementary Table 1.

### Overall prognostic value of IL-6 in mCRC

**Figure 1:**
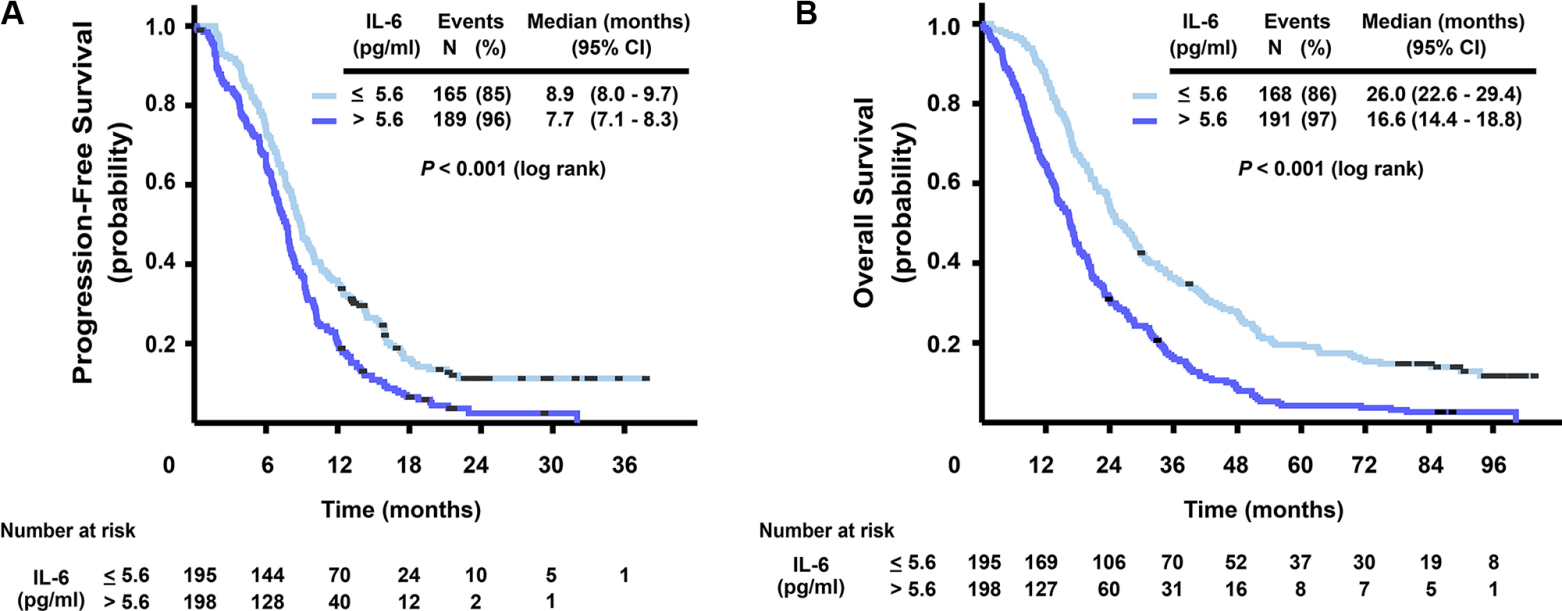
Kaplan-Meier curves for progression-free survival (A) and overall survival (B), stratified by IL-6 serum level (dichotomized at median, ≤ 5.6, > 5.6 pg/ml)

High pretreatment levels of serum IL-6 were strongly associated with impaired survival. Figure 1 shows that patients with high versus low IL-6 levels (dichotomized at median 5.6 pg/ml) had a median PFS of 7.7 versus 8.9 months, respectively (HR = 1.54, 95% CI 1.25–1.91, *P* < 0.001), and a median OS of 16.6 versus 26.0 months (HR = 1.92, 95% CI 1.56–2.37, *P* < 0.001). The prognostic role of baseline levels of IL-6 on OS was also confirmed in a model adjusting for other prognostic markers and clinical characteristics (CEA, mutation status, ALP and WHO performance status). Adding treatment arm or interaction between treatment arm and IL-6 in the analysis did not provide extra information. Sensitivity analysis with other cut-offs than the median for IL-6 gave essentially the same results.

### Prognostic role of IL-6 in subgroups according to *RAS* and *BRAF* mutation status

The data indicated that the prognostic role of IL-6 differed depending on the *RAS* and *BRAF* mutation status, with a statistically significant interaction in unadjusted and adjusted analyses, *P* = 0.004 (Table 2). There was a much larger relative reduction in survival associated with high IL-6 in the *BRAF*-mutated group, as analyzed with Cox regression for OS (HR = 4.05, 95% CI 2.13–7.71), compared to the double wild-type (HR = 1.44, 95% CI 1.00–2.06) and the *RAS*-mutated groups (HR = 1.23, 95% CI 0.88–1.72). Median PFS and OS for patients with high versus low IL-6, sub-classified according to *RAS* and *BRAF* mutation status are summarized in Table 3.

**Table 2:** Multivariable cox regression analysis for OS including interaction term for IL-6 and *RAS* and *BRAF* mutation status in 364 patients

Variable		Adjusted analysis, IL-6		
		HR	95% CI	*P*-value
**Mutation status**	IL-6 pg/ml			
*RAS / BRAF* WT (*n* = 154)	≤ 5.8 > 5.8	1 1.44	1.00–2.06	0.004*
*RAS* Mut (*n* = 167)	≤ 5.8 > 5.8	1 1.23	0.88–1.72	
*BRAF* Mut (*n* = 43)	≤ 5.8 > 5.8	1 4.05	2.13–7.71	
CEA mg/L < 5 (*n* = 70) ≥ 5 (*n* = 294)		1 1.68	1.23–2.30	< 0.001
ALP Normal (*n* = 195) Abnormal (*n* = 169)		1 1.58	1.24–2.01	< 0.001
WHO performance status 0 (*n* = 235) 1 (*n* = 105) 2 (*n* = 24)		1 1.29 2.70	1.00–1.66 1.71–4.26	< 0.001 0.048 < 0.001

Abbreviations: Mut, mutant; WT, wild- type; OS, overall survival; CEA, Carcinoembryonic antigen; ALP, Alkaline phosphatase

* interaction *P*.

**Table 3:** Median PFS and OS, subclassified according to *RAS* and *BRAF* mutation status in 393 patients

	PFS						OS		
		*RAS/BRAF* WT		*RAS* Mut		*BRAF* Mut	*RAS/BRAF* WT	*RAS* Mut	*BRAF* Mut
	*n* (%)	Median (months) 95% CI	*n* (%)	Median (months) 95% CI	*n* (%)	Median (months) 95% CI	Median (months) 95% CI	Median (months) 95% CI	Median (months) 95% CI
**IL-6, pg/ml** ≤ 5.6 > 5.6	79 (47.0) 89 (53.0)	10.9 (8.5–13.3) 8.4 (7.4–9.5) *P* < 0.001	97 (54.2) 82 (45.8)	8.2 (7.5–8.8) 7.8 (6.8–8.8) *P* = 0.364	19 (41.3) 27 (58.7)	9.0 (3.6–14.3) 4.6 (3.0–6.3) *P* < 0.001	35.3 (25.3–45.3) 20.5 (18.8–22.3) *P* < 0.001	23.4 (19.3–27.5) 16.6 (14.2–19.1) *P* = 0.002	17.0 (6.9–27.0) 6.9 (4.7–9.2) *P* < 0.001
**CRP, mg/L** ≤ 10 11–30 31–60 > 60	69 (41.1) 49 (29.2) 26 (15.5) 24 (14.3)	10.9 (7.9–13.9) 9.3 (8.2–10.4) 9.2 (6.5–11.9) 7.3 (5.6–9.0) *P* = 0.005	83 (46.4) 48 (26.8) 20 (11.2) 28 (15.6)	8.3 (7.7–8.9) 6.7 (5.6–7.9) 7.1 (4.6–9.5) 6.8 (4.7–9.0) *P* = 0.089	21 (45.7) 10 (21.7) 7 (15.2) 8 (17.4)	6.9 (4.6–9.2) 5.4 (2.7–8.1) 5.8 (1.2–10.2) 1.8 (0–4.5) *P* = 0.054	33.8 (26.2–41.4) 22.6 (12.3–32.9) 20.5 (15.4–25.6) 12.8 (9.2–16.3) *P* < 0.001	20.5 (16.0–24.9) 20.6 (14.8–26.4) 16.2 (11.7–20.7) 14.2 (5.2–23.1) *P* < 0.009	14.1 (8.4–19.9) 8.1 (5.1–11.1) 7.6 (4.2–11.0) 3.8 (2.1–5.5) *P* < 0.008

Abbreviations: PFS, progression-free survival; OS, overall survival; IL-6, Interleukin 6; CRP, C-reactive protein; WT, wild- type; Mut, mutant.

### Comparison of inflammatory markers and correlation with IL-6

The prognostic role of the different markers of SIR studied here, i.e. mGPS, dNLR, platelets, and CRP, in terms of PFS and OS, was examined. The results showed that for all these SIR markers high levels were associated with poor outcome, and no statistically significant differences in their prognostic value were detected (Table 4). Only CRP was used as a SIR biomarker in the further analyses. Supplementary Figure 1 shows a positive correlation between values of serum CRP and IL-6 (*r* = 0.66, *P* < 0.001), indicating that about 40% of the variability in the CRP values could be accounted for by IL-6. The frequency distribution of CRP and IL-6 levels was similar in the different mutation subgroups (Table 3). Neither were there any major differences in the distribution of platelets, dNLR, and mGPS overall or for the different subgroups, see Supplementary Table 2.

**Table 4:** Prognostic information in terms of PFS and OS of different markers of systemic inflammatory response (SIR) in 374 patients

	*n* (%)	PFS			OS		
		HR	95%CI	*P*-value	HR	95% CI	*P*-value
CRP (mg/L) ≤ 10 11–30 31–60 > 60	165 (44.1) 100 (26.7) 51 (13.6) 58 (15.5)	1 1.63 1.45 1.82	1.25–2.12 1.04–2.01 1.33–2.49	< 0.001 < 0.001 0.027 < 0.001	1 1.38 1.88 2.45	1.06–1.79 1.35–2.62 1.79–3.35	< 0.001 0.016 < 0.001 < 0.001
Platelets(10^9^/ L) ≤ 400 > 400	266 (71.1) 108 (28.9)	1 1.60	1.27–2.02	< 0.001	1 1.85	1.47–2.34	< 0.001
mGPS 0 1 2	165 (44.1) 166 (44.4) 43 (11.5)	1 1.55 1.99	1.23–1.95 1.40–2.81	< 0.001 < 0.001 < 0.001	1 1.60 2.16	1.27–2.01 1.52–3.06	< 0.001 < 0.001 < 0.001
dNLR ≤ 2.1 > 2.1	187 (50.0) 187 (50.0)	1 1.56	1.25–1.93	< 0.001	1 1.68	1.35–2.08	< 0.001

Abbreviations: CRP, C-reactiv protein; mGPS, Modified Glasgow Prognostic Score: 0 = CRP ≤ 10 mg/L (independent of the albumin level), 1 = CRP > 10 mg/L and albumin ≥ 35 g/L, 2 = CRP > 10 mg/L and albumin < 35 g/L; dNLR, Derived Neutrophil to Lymphcyte Ratio.

### Prognostic value of CRP

**Figure 2:**
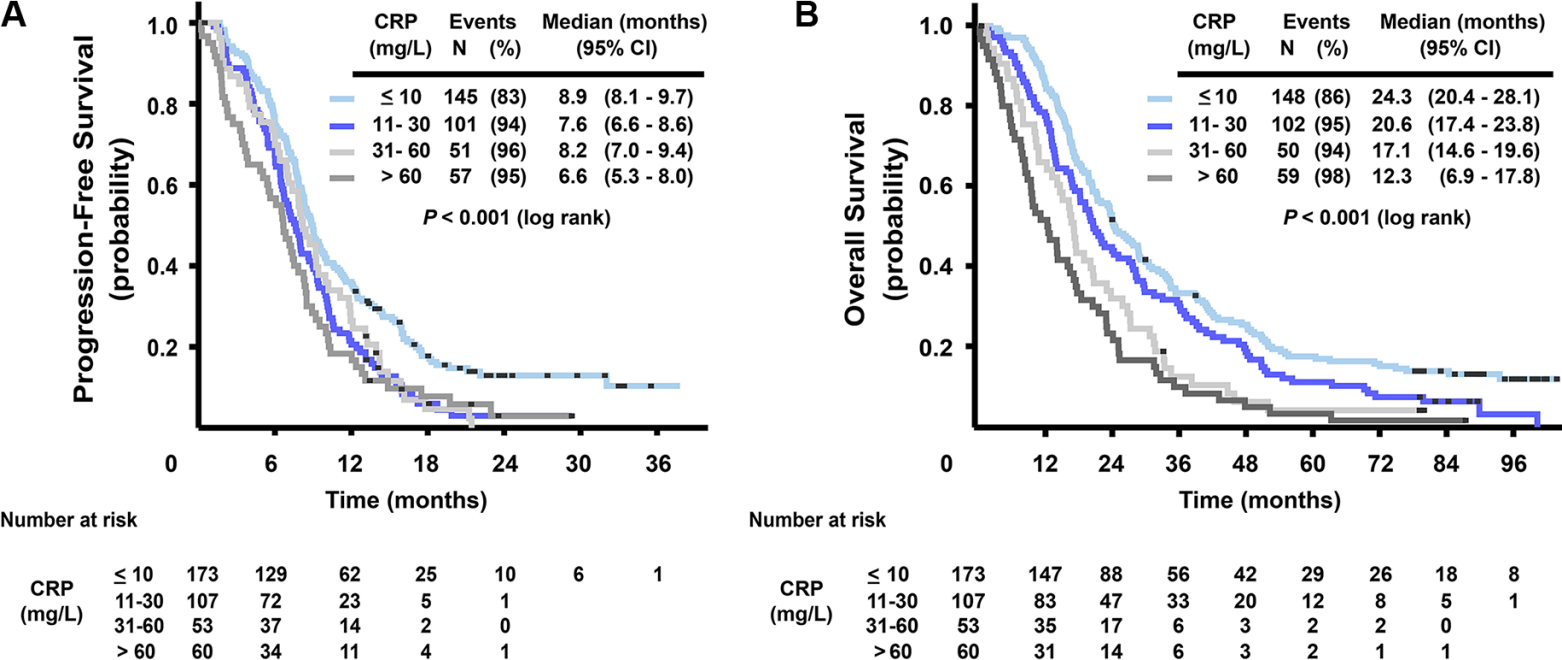
Kaplan-Meier curves for progression-free survival (A) and overall survival (B), stratified by CRP serum level (≤ 10, 11–30, 31–60 and > 60 mg/L)

High levels of CRP were associated with short survival. Figure 2 shows that in the four categories of baseline serum CRP level (≤ 10, 11–30, 31–60 and > 60 mg/L), median PFS was 8.9, 7.6, 8.2, and 6.6 months, respectively (log rank test, *P* < 0.001) and median OS was 24.3, 20.6, 17.1, and 12.3 months, respectively (log rank test, *P* < 0.001). The prognostic role of baseline serum CRP in terms of OS was also confirmed in a model adjusted for other prognostic markers and clinical characteristics (CEA, mutation status, ALP and WHO performance status) (HR 1.16, 95% CI 1.03–1.30, *P* = 0.015), see Supplementary Table 3. Adding treatment arm in the analysis gave no additional information.

Median PFS and OS for patients with different CRP levels according to mutation status are summarized in Table 3. OS for patients with CRP values under 10 mg/L compared to CRP values over 60 mg/L were 33.8 versus 12.8 months in the wild-type group, 20.5 versus 14.2 months in the *RAS* mutated group and 14.1 versus 3.8 months in the *BRAF* mutated group. Although these relationships between serum levels of CRP and treatment outcome largely followed the same pattern as for IL-6 in the subgroups of *RAS* and *BRAF* mutation status, the interaction between mutation status and different CRP levels was not statistically significant.

### Changes in CRP and IL-6 over time

Having shown that baseline levels of both serum IL-6 and CRP have statistically significant impact on survival, we further explored whether changes in IL-6 and CRP over the first 8 weeks of treatment would improve the prognostic value of the markers. Mutation status did not significantly affect the distribution of change in either inflammatory marker. The distribution of change in CRP was also similar in the three arms, but the proportion of patients with a reduction in IL-6 was slightly higher in arm A (Supplementary Table 4). Several exploratory analyses with different measures of changes over time were performed. None of the models including changes in serum IL-6 or CRP over 8 weeks improved prediction of PFS or OS over models including baseline IL-6 or CRP only (Supplementary Table 5).

## DISCUSSION

This study adds to the evidence of a role of inflammatory processes in cancer, as it demonstrates that high levels of inflammation biomarkers predict markedly impaired survival in a large cohort of patients with mCRC. Furthermore, it provides more results to support the involvement of IL-6 in tumor-promoting inflammation in this malignancy, and the data also suggest that IL-6 interacts differentially with the tumorigenic mechanisms of mutated *BRAF* and *RAS* in CRC. Finally, the study demonstrates that CRP, which is easily available in routine clinical practice, is a useful prognostic biomarker in this disease.

The role of IL-6 in inflammation and in the pathogenesis of various human diseases is well documented [25, 26, 40]. In cancer, IL-6 is produced by several cells in a tumor and can exert its pleiotropic effects on both malignant and stromal cells [30, 41]. This is the case for both sporadic and inflammation-associated CRC [7, 19, 30, 34, 41]. In the present study of patients with mCRC, we found that high serum concentrations of IL-6 were associated with markedly impaired prognosis.

Furthermore, the finding that a high level of IL-6 was associated with a particularly large relative reduction in survival in patients with mutated *BRAF,* unlike mutated *RAS,* is worth noting. Although the molecular explanation for this is not known, it might reflect differences in the way IL-6 interacts with the tumorigenic mechanisms activated by the mutated *BRAF* and mutated *RAS*. The serine/threonine kinase BRAF, which is activated dramatically by the V600E mutation [42], is a selective activator of the MEK/ERK pathway, a major mediator of cell cycle entry. IL-6 primarily stimulates pro-inflammatory pathways, notably JAK/STAT signaling, which exerts several potentially tumor-promoting effects, including angiogenesis and enhanced invasive capacity [33, 40]. It is likely that these complementary mechanisms may form a basis for synergism. In contrast, mutated *RAS* can mediate enhanced activity in several pathways [43], leading to stimulation not only of the ERK cascade but also the PI3K/Akt and other pathways, with downstream mechanisms that may enhance inflammatory processes, and it is conceivable that a pro-inflammatory stimulus from IL-6 may have a somewhat weaker impact on a *RAS-*driven malignancy.

A major aim of this study was to examine the prognostic potential of easily available markers of inflammation in mCRC. It was found that the level of CRP was a strong prognostic predictor in mCRC, in agreement with data recently reported by others [38]. Regarding survival, CRP was an equally good marker as dNLR, mGPS and platelets. The median OS was 33.8 months in patients whose tumors were double wild-type and who had normal CRP and 12.8 months in the highest CRP group. Corresponding results for mutated *RAS* and mutated *BRAF* groups were 20.5 versus 14.2 and 14.1 versus 3.8 months, respectively. However, the interaction between *RAS* or *BRAF* mutation status and CRP value, unlike IL-6, was not statistically significant. This and the fact that the correlation between IL-6 and CRP, although statistically significant, was not very strong, indicate that CRP likely reflects a broader measure of the inflammatory status than IL-6.

The marked difference in OS for high versus low IL-6 or CRP despite relatively small differences in PFS is consistent with data indicating a special role of inflammation in advanced and cachectic cancer [5, 23]. Baseline serum IL-6 and CRP levels were better predictors of survival than changes in IL-6 and CRP levels during treatment. This probably reflects distinct phenotypes from early stages of the tumor development.

The inflammatory burden is likely responsible for clinical effects such as weight loss and poor performance status [9, 22, 23]. Like some of the other established markers of SIR, CRP has the advantage of being inexpensive, and it can be measured routinely both in daily clinical practice and in study settings, making it easily accessible, and, in addition, it is also readily and precisely quantifiable [36]. Since increasing CRP values confer an increasingly worse prognosis independent of other well-known prognostic markers, CRP adds important clinical value. Combining information on CRP and mutation status provides improved prognostic information that may guide treatment decisions concerning the need for aggressive first-line treatment for some patients. On the other hand, some patients with *BRAF* mutation combined with elevated CRP have a very poor prognosis and might have limited benefit from chemotherapy.

The main strength of this study is that it is large and analyzes multiple markers of SIR in a cohort of prospectively collected data from patients with mCRC. Most previous reports of systemic inflammation come from cohorts of cancer patients treated with surgery, and only a few reports focus on patients with unresectable metastatic cancer. The size of this study provides the opportunity to compare different markers of SIR and also allows analysis of the prognostic role of IL-6 and inflammation as related to mutation status. However, there are also several limitations, as not all variables were available for all patients, and data on possible ongoing infections are lacking.

In conclusion, serum IL-6 and CRP are good prognostic biomarkers in patients with mCRC independent of the *RAS* and *BRAF* mutation status. The effect of IL-6 was particularly pronounced in patients with *BRAF* mutation. Combining the prognostic information of CRP and IL-6 with knowledge of the *RAS* and *BRAF* mutation status may help in treatment decisions. It is conceivable that patients with high serum IL-6 might benefit from treatment interrupting the inflammation, and drugs targeting the IL-6/IL-6R axis might be a future treatment option in CRC [6, 34, 44, 45].

## MATERIALS AND METHODS

### Patients

The randomized NORDIC-VII study [39] investigated the effects of combining cetuximab with a regimen of bolus 5-flourouracil (5-FU)/folinic acid (FA) and oxaliplatin (Nordic FLOX) [46] in first-line therapy of mCRC. Patients were randomly assigned to receive standard Nordic FLOX, cetuximab and FLOX, or cetuximab combined with intermittent FLOX. Since there was no statistically significant difference in outcome between the treatment arms, neither in the original analysis [39] nor in subsequent updated overall survival data until April 30, 2014 (unpublished data), the present study used the whole patient population across the different treatments in NORDIC-VII.

*RAS* and *BRAF* mutation status was available for 393 patients. Of these 364 patients were eligible for the multivariable analysis of clinical and prognostic markers, whereas 374 patients were eligible for the comparison of different markers of SIR with available albumin, CRP, platelets, neutrophils and leukocytes at baseline. Measures of both serum IL-6 and CRP at baseline and at week 8 were available for 393 and 275 patients, respectively. Mutation status (*RAS, RAF*, double wild-type) was obtained for the whole study population.

### Collection of blood samples and IL-6 measurement

WBC, ANC, platelet count and hemoglobin were measured at baseline and before each treatment cycle. Albumin was analysed at baseline and every second cycle and CRP was measured at baseline, after one week and then before every cycle for four cycles and every fourth cycle thereafter. The analyses were conducted according to current practice at each participating hospital. Fresh-frozen serum/plasma samples were collected at baseline, after the first week of cycle 1, after the second week of cycle 1 and after the second week of cycle 4. Serum concentration of IL-6 was determined in serum samples stored at minus 80°C by a commercially available human IL-6 high-sensitive enzyme-linked immunosorbent assay (ELISA, Quantikine HS, high sensitive, R&D Systems, Abingdon, Oxon, UK) [47].

### Mutation analyses of *KRAS*, *NRAS* and *BRAF*

Genomic DNA was extracted from formalin-fixed paraffin-embedded (FFPE) 10 μm tissue sections with 65% to 70% (median) tumour cells using QIAamp DNA Micro Kit (Cat.56304, Quiagen). Tumour DNA was screened for the presence of the *KRAS* mutations Q61H, Q61L, Q61R, K117X (K117N 351A > C, K117N 351A > T, K117R, K117E) and A146X (A146T, A146P, A146V) using the *KRAS* Mutation Analysis Kit for Real-Time PCR (exons 2, 3 and 4) by EntroGen. The *NRAS* mutations G12C, G12D, G12S, G13V, G13R, Q61K, Q61R, Q61L, Q61H and A146T were analyzed using the *NRAS* Mutation Analysis kit (EntroGen). The mutation detection assays and the analysis of the results were performed in accordance with the manufacturer's instructions. Input in the *KRAS* and *NRAS* assays were 10 ng and 20 ng DNA, respectively. Analyses for the *BRAF* V600E mutation was performed as described by Hamfjord et al. [48]

### Markers of SIR

dNLR, platelet counts, mGPS, and CRP were chosen as clinically useful markers of SIR. dNLR was applied with the assumption that white blood cell counts is made up primarily of lymphocytes and neutrophils, and that the white cell count minus neutrophil count would be quite similar to the lymphocyte count [37]. Separate analyses of the prognostic effect of each different inflammatory marker on PFS and OS were performed. Patients were grouped into four CRP categories (≤ 10, 11–30, 31–60, > 60 mg/L), based upon current reference cut-off values in colon cancer [36]. dNLR was dichotomized at the median value 2.1, as no consensus regarding cut-off values exists in the literature, but in accordance with previous publications [37]. Platelet counts were dichotomized at below or above 400 ·109/ L. mGPS was classified from 0-2, 0 defined as CRP ≤ 10 mg/L (independent of the albumin level), 1 defined as CRP > 10 mg/L and albumin ≥ 35 g/L, and 2 defined as CRP > 10 mg/L and albumin < 35 g/L [49]. IL-6 levels were dichotomized at the median 5.6 pg/ml, as no consensus exists in the literature concerning cut-off value [30]. Reported median serum IL-6 in healthy subjects was 1.4pg/ml [47]. The results of IL-6 and mutation analyses are presented in accordance with REMARK (Reporting Recommendations for Tumor Marker Prognostic Studies) guidelines [50]. The remaining analyses were conducted according to current practice at each participating hospital.

### Ethics

The NORDIC-VII study (http://clinicaltrials.gov/show/NCT00145314) was approved by the national ethics committees and governmental authorities in each country and conducted in accordance with the Declaration of Helsinki. All patients gave written informed consent.

### Statistical analysis

The statistical analyses were performed using IBM SPSS (version 18.0. Chicago: SPSS Inc.). Demographic data were described with median and range (continuous variables) and with proportion and percentages (categorical variables). The prognostic values of different categories of IL-6 and CRP values were assesed by Kaplan-Meier plots, log-rank test, and Cox proportional hazards model. Separate analyses of the effect of IL-6, CRP, CEA, ALP, WHO performance status, mutation status, platelets, WBC, ANC, metastatic site, number of metastatic sites, sex, tumor location, treatment arm and age were performed. Only variables statistically significant in these analyses were included in the multivariable analyses and models were restricted to include statistically significant variables only. Correlation between IL-6 and CRP was estimated with Pearson's r using log-transformed values to ensure an approximately linear association. An interaction term was included in the Cox model to explore the effect of mutation status on the prognostic effect of IL-6 and CRP, respectively. Several different measures of change in CRP and IL-6 over 8 weeks; absolute and relative difference, as well as various categorizations were explored and compared using landmark analysis. Model fit was compared by likelihood ratio tests, and the model finally chosen was the one resulting in the best fit.

## SUPPLEMENTARY MATERIALS FIGURES AND TABLES


