# Supplementary material for: Interleukin-6 and C-reactive protein as prognostic biomarkers in metastatic colorectal cancer
Source: Oncotarget. 2016 Oct 12;7(46):75013–22. doi: 10.18632/oncotarget.12601 (PMC5342719; doi:10.18632/oncotarget.12601)
Supplement: Supplementary file 1 [file oncotarget-07-75013-s001.pdf]

Interleukin-6 and C-reactive protein as prognostic biomarkers in metastatic colorectal cancer

Supplementary Materials

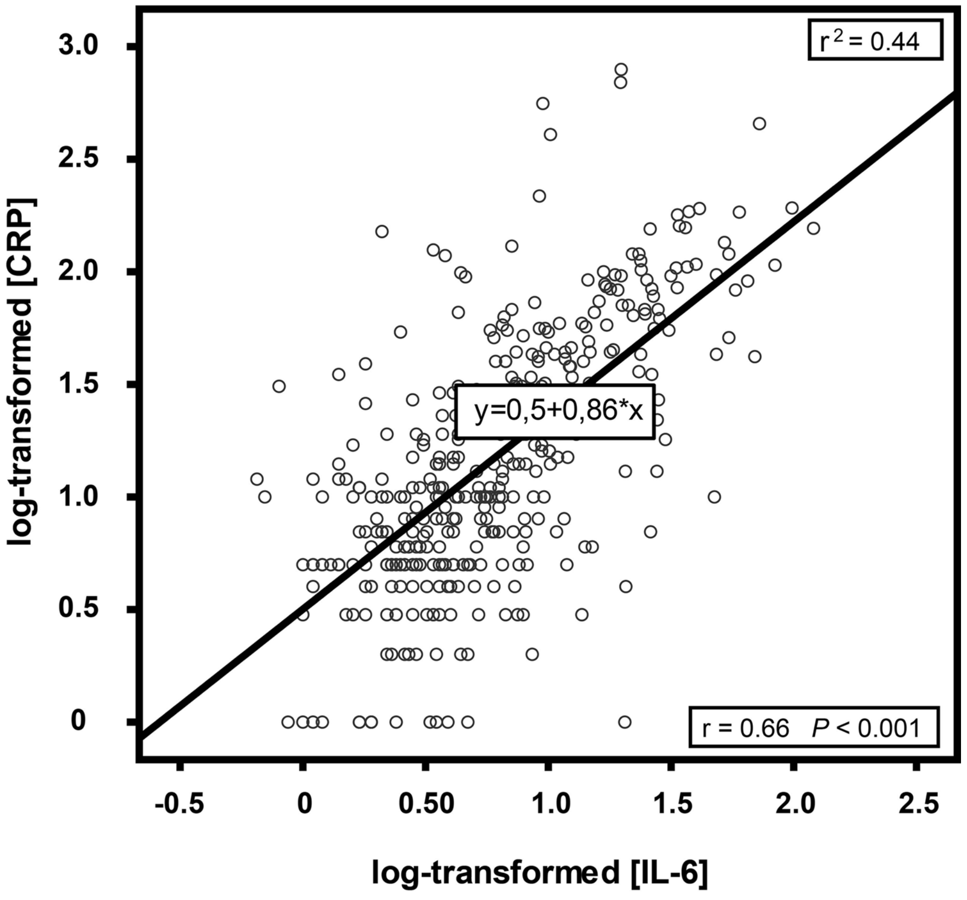

Supplementary Figure S1: Correlation between log-transformed IL-6 and log-transformed CRP.

Supplementary Table S1: Distribution of inflammatory markers by tumor burden and site of metastases in 393 patients

	IL-6 pg/ml		CRP mg/L			
	≤ 5.6	> 5.6	≤ 10	11–30	31–60	> 60
**Number of metastatic sites**						
1	61 (49.6)	62 (50.4)	51 (41.5)	41 (33.3)	16 (13.0)	15 (12.2)
> 1	134 (49.6)	136 (50.4)	122 (45.2)	66 (24.4)	37 (13.7)	45 (16.7)
**Metastatic site**						
Liver only	38 (46.3)	44 (53.7)	28 (34.1)	30 (36.6)	14 (17.1)	10 (12.2)
Liver+ other	100 (48.1)	108 (51.9)	80 (38.5)	57 (27.4)	32 (15.4)	39 (18.8)
Non liver	57 (55.3)	46 (44.7)	65 (63.1)	20 (19.4)	7 (6.8)	11 (10.7)

Abbreviations: CRP, C-reactive protein; IL-6, Interleukin 6.

Supplementary Table S2: Frequency of different markers of Systemic inflammatory response (SIR), total and classified by *RAS* and *BRAF* mutation status

Variable	Total<br>*n* = 374<br>*n* (%)	*RAS* and *BRAF* WT<br>*n* = 159 (42.5)<br>*n* (%)	*RAS* Mut<br>*n* = 171 (45.7)<br>*n* (%)	*BRAF* Mut<br>*n* = 44 (11.8)<br>*n* (%)
**CRP (mg/L)**				
≤ 10	165 (44.1)	66 (41.5)	79 (46.2)	20 (45.5)
11–30	100 (26.7)	44 (27.7)	46 (26.9)	10 (22.7)
31–60	51 (13.6)	26 (16.4)	18 (10.5)	7 (15.9)
> 60	58 (15.5)	23 (14.5)	28 (16.4)	7 (15.9)
**Platelets** (10^9/ L)				
≤ 400	266 (71.1)	117 (73.6)	120 (70.2)	29 (65.9)
> 400	108 (28.9)	42 (26.4)	51 (29.8)	15 (34.1)
**mGPS**				
0	165 (44.1)	66 (41.5)	79 (46.2)	20 (45.5)
1	166 (44.4)	73 (45.9)	73 (42.7)	20 (45.5)
2	43 (11.5)	20 (12.6)	19 (11.1)	4 (9.1)
**dNLR**				
≤ 2.1	187 (50.0)	87 (54.7)	77 (45.0)	23 (52.3)
> 2.1	187 (50.0)	72 (45.3)	94 (55.0)	21 (47.7)

Abbreviations: Mut, mutant; WT, wild- type; CRP, C-reactiv protein; mGPS, Modified Glasgow Prognostic Score: 0 = CRP ≤ 10 mg/L (independent of the albumin level), 1 = CRP > 10 mg/L and albumin ≥ 35 g/L, 2 = CRP > 10 mg/L and albumin < 35 g/L; dNLR, Derived Neutrophil to Lymphcyte Ratio.

Supplementary Table S3: Association between overall survival and clinical variables and other prognostic markers in 364 patients

	n (%)	Unadjusted analysis			Adjusted analysis, CRP		
		HR	95% CI	*P*-value	HR	95% CI	*P*-value
**CRP mg/L**							
≤ 10	156 (42.9)	1.35	1.23–1.49	< 0.001	1.16	1.03–1.30	0.015
11–30	100 (27.5)						
31–60	50 (13.7)						
> 60	58 (15.9)						
**IL-6 pg/ml**							
≤ 5.8	182 (50.0)	1					
> 5.8	182 (50.0)	1.86	1.50–2.32	< 0.001			
***RAS/BRAF* Mutation status**				< 0.001			< 0.001
double WT	154 (42.3)	1			1		
RAS Mut	167 (45.9)	1.21	0.96–1.53	0.105	1.38	1.09–1.74	0.008
BRAF Mut	43 (11.8)	2.51	1.78–3.56	< 0.001	3.53	2.44–5.11	< 0.001
**CEA mg/L**							
< 5	70 (19.2)	1			1		
≥ 5	294 (80.8)	1.87	1.39–2.52	< 0.001	1.94	1.41–2.66	< 0.001
**ALP**							
Normal	195 (53.6)	1			1		
Abnormal	169 (46.4)	1.98	1.59–2.47	< 0.001	1.52	1.18–1.96	0.001
**WHO performance status**				< 0.001			0.002
0	235 (64.6)	1			1		
1	105 (28.8)	1.64	1.29–2.09	< 0.001	1.25	0.97–1.61	0.083
2	24 (6.6)	3.63	2.36–5.58	< 0.001	2.34	1.45–3.76	< 0.001
**Platelets 10^9/L**							
≤ 400	256 (70.3)	1					
> 400	108 (29.7)	1.83	1.45–2.32	< 0.001			
**WBC 10^9/L**							
≤ 10	292 (80.2)	1					
> 10	72 (19.8)	1.39	1.06–1.81	0.016			
**ANC 10^9/L**							
≤ 8	315 (86.5)	1					
> 8	49 (13.5)	1.87	1.38–2.54	< 0.001			
**Metastatic site**				0.103			
Non liver	97 (26.6)	1					
Liver only	77 (21.2)	1.04	0.76–1.43	0.805			
Liver +other	190 (52.2)	1.29	0.99–1.67	0.057			
**Number of metastatic sites**							
1	116 (31.9)	1					
> 1	248 (68.1)	1.18	0.94–1.49	0.156			
**Sex**							
Male	221 (60.7)	1					
Female	143 (39.3)	1.06	0.85–1.32	0.614			
**Location**							
Colon	218 (59.9)	1					
Rectum	146 (40.1)	0.84	0.68–1.05	0.121			
**Treatment arm**				0.520			
A	111 (30.5)	1					
B	132 (36.3)	1.04	0.80–1.36	0.766			
C	121 (33.2)	1.16	0.89–1.52	0.275			
**Age**		1.00	0.99–1.01	0.718			

Abbreviations: CRP, C-reactive protein; IL-6, Interleukin 6; Mut, mutant; WT, wild- type; CEA, Carcinoembryonic antigen; ALP, Alkaline phosphatase; WBC, White blood cell; ANC, Absolute neutrophil count; Treatment arm A, Nordic FLOX;Treatment arm B, cetuximab and Nordic FLOX; Treatment arm C, cetuximab and intermittent Nordic FLOX.

Supplementary Table S4: Distribution of changes in inflammatory markers

	CRP *n* (%)			IL-6 *n* (%)		
	Reduction	No change	Increase	Reduction	No change	Increase
**Treatment arm**						
A	53 (58)	25 (28)	13 (14)	36 (40)	26 (29)	29 (32)
B	58 (64)	12 (13)	21 (23)	30 (33)	14 (15)	47 (52)
C	59 (63)	12 (13)	22 (24)	29 (31)	17 (18)	47 (51)
***RAS/BRAF* Mutation status**						
Double WT	44 (36)	26 (21)	52 (43)	44 (36)	26 (21)	52 (43)
RAS Mut	44 (35)	26 (21)	55 (44)	44 (35)	26 (21)	55 (44)
BRAF Mut	7 (25)	5 (18)	16 (57)	7 (25)	5 (18)	16 (57)

Mutation status does not significantly affect the distribution of change in either inflammatory marker. The distribution of CRP is also similar in the three treatment arms, but the proportion with a reduction in IL-6 is slightly higher in arm A.

Abbreviations: CRP, C-reactive protein; IL-6, Interleukin 6; Treatment arm A, Nordic FLOX;Treatment arm B, cetuximab and Nordic FLOX; Treatment arm C, cetuximab and intermittent Nordic FLOX; Mut, mutant; WT, wild- type.

Supplementary Table S5: Prognostic effect of change in inflammatory markers on overall survival from week 8 in different adjusted[1] models

	HR (95% CI)		
	**Baseline only**	**Change only**	**Both baseline and change**
**CRP**			
Baseline	1.22 (1.06–1.41)	–	1.22 (1.04–1.43)
Reduction	–	1	1
No change	–	0.70 (0.48–1.01)	0.84 (0.56–1.27)
Increase	–	0.97 (0.68–1.37)	1.16 (0.79–1.70)
**IL-6**			
Baseline	1.33 (1.00–1.76)	–	1.42 (1.04–1.95)
Reduction	–	1	1
No change	–	0.85 (0.59–1.24)	1.00 (0.67–1.48)
Increase	–	1.07 (0.78–1.45)	1.26 (0.89–1.78)

There is no prognostic effect of change in CRP or IL-6, neither when included alone in the adjusted model, nor when added to an adjusted model including the baseline value of the inflammatory marker.

[1]Adjusted for WHO performance status, ALP, CEA, *RAS/BRAF* mutation. For simplicity the interaction between baseline IL-6 and mutation status has been omitted.

Abbreviations: CRP, C-reactive protein; IL-6, Interleukin 6;
